# Microfluidic particle dam for direct visualization of SARS-CoV-2 antibody levels in COVID-19 vaccinees

**DOI:** 10.1126/sciadv.abn6064

**Published:** 2022-06-03

**Authors:** Minghui Wu, Siying Wu, Gaobo Wang, Wengang Liu, Lok Ting Chu, Tianyi Jiang, Hoi Kwan Kwong, Hiu Lam Chow, Iris Wai Sum Li, Ting-Hsuan Chen

**Affiliations:** 1 Department of Biomedical Engineering, City University of Hong Kong, Hong Kong Special Administrative Region, China.; 2 HKU-Pasteur Research Pole, The University of Hong Kong, Pokfulam, Hong Kong Special Administrative Region, China.; 3 City University of Hong Kong Shenzhen Research Institute, Shenzhen, China.

## Abstract

Various COVID-19 vaccines are currently deployed, but their immunization varies and decays with time. Antibody level is a potent correlate to immune protection, but its quantitation relies on intensive laboratory techniques. Here, we report a decentralized, instrument-free microfluidic device that directly visualizes SARS-CoV-2 antibody levels. Magnetic microparticles (MMPs) and polystyrene microparticles (PMPs) can bind to SARS-CoV-2 antibodies simultaneously. In a microfluidic chip, this binding reduces the incidence of free PMPs escaping from magnetic separation and shortens PMP accumulation length at a particle dam. This visual quantitative result enables use in either sensitive mode [limit of detection (LOD): 13.3 ng/ml; sample-to-answer time: 70 min] or rapid mode (LOD: 57.8 ng/ml; sample-to-answer time: 20 min) and closely agrees with the gold standard enzyme-linked immunosorbent assay. Trials on 91 vaccinees revealed higher antibody levels in mRNA vaccinees than in inactivated vaccinees and their decay in 45 days, demonstrating the need for point-of-care devices to monitor immune protection.

## Teaser

Microfluidic particle dam visualizes antibody levels in COVID-19 vaccinees.

## INTRODUCTION

The coronavirus disease 2019 (COVID-19) pandemic has severely affected public health and the economy (*1*–*4*). According to the World Health Organization (WHO), globally, there are more than 247 million confirmed cases and about 5.02 million deaths at the beginning of November 2021. Regrettably, the virus continues to adapt in humans and spreads rapidly such that the pandemic is not validly controlled yet. To slow down the spread within communities, administrative measures including territory lockdown, cross-border restrictions, and mandatory isolations/quarantines have been implemented to minimize people’s movements and contacts. However, it also retards the economies and causes fears and panic in public (*3*, *4*).

As the ultimate hope to stop the COVID-19 pandemic, various COVID-19 vaccines have been deployed worldwide. At the end of November 2021, 54% of the world population has received at least one dose (*5*). Upon vaccination, the immunoglobulin G (IgG) and immunoglobulin M (IgM) antibody level against severe acute respiratory syndrome coronavirus 2 (SARS-CoV-2) increases in 10 days (*6*), and their amount correlates with virus neutralization (*7*, *8*). As antibodies readily available in blood could theoretically neutralize viruses in the circulation more readily than those without, serum antibody levels, including both neutralizing antibody and binding antibody, have been proposed as a potent correlate of immune protection (*8*–*10*). In Hong Kong, two types of vaccines (mRNA in lipid nanoparticle vaccine from BioNTech and inactivated whole virion vaccine from Sinovac) are currently available. However, a recent report has shown that immunogenicity varies in several orders of magnitude depending on vaccine types (*11*), which implies varied efficacy. More critically, the antibody level decays rapidly (*12*), suggesting decaying immune protection with time. Thus, individuals have no clue about the status of protection by vaccination and its durability.

Measurement of antibody level provides a direct index for evaluation of an individual’s immune status. Conventional immunoassay assays such as enzyme-linked immunosorbent assay (ELISA) and chemiluminescence immunoassay have been the standard tests for antibody detection (*13*–*17*). However, these assays require professional experts in accredited laboratories to carry out the procedures and interpret results, and they are expensive and technically intensive. Because of the overwhelmed medical facilities during the pandemic, rapid tests such as lateral flow immunoassay (LFIA) have been used at border control, primary care, and community settings for their superior simplicity in operation and signal reporting (*18*–*22*). However, LFIA uses the appearance of color bands that provide only qualitative results, i.e., yes/no. Moreover, LFIA has been reported to be insensitive and ambiguous, except when used in symptomatic and severely diseased patients (*23*–*27*).

To achieve quantitative measurement while maintaining a simple and user-friendly interface, attempts have been made to conduct immunoassays in miniaturized devices (*28*) using principles such as ELISA (*29*–*31*), electrochemistry (*32*, *33*), surface plasmon resonance (*34*), and fluorescence (*35*–*38*). However, signal quantification requires additional detection modules, e.g., optical fibers, light source, amperemeters, or photodetectors, for measuring optical absorbance or fluorescence intensity. Customized settings are needed during miniaturization, making them still laboratory based, and they do not necessarily lead to simplicity.

**Fig. 1.**
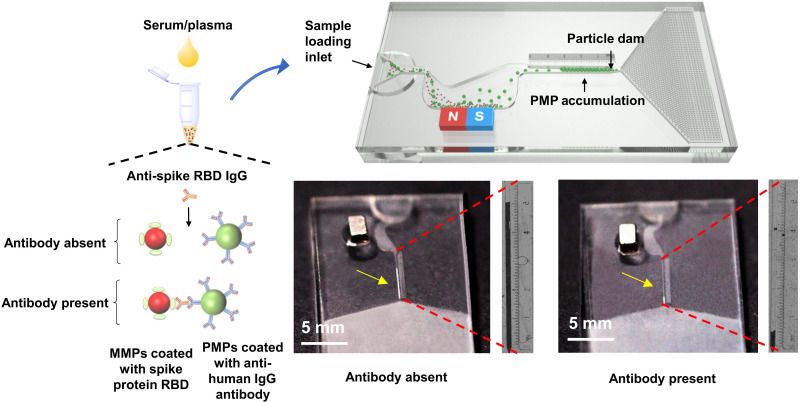
Schematic illustration of the working principle. The IgG antibody against SARS-CoV-2 spike protein receptor-binding domain (anti-spike RBD IgG) in serum/plasma simultaneously binds to magnetic microparticles (MMPs) coated with SARS-CoV-2 spike protein RBD and polystyrene microparticles (PMPs) coated with anti-human IgG antibody, forming MMPs-antibodies-PMPs. After loading to a capillary-driven microfluidic chip, the MMPs and MMPs-antibodies-PMPs are automatically separated in a magnetic separator. At the same time, free PMPs continue to flow until they are trapped at a particle dam with a narrowing nozzle. Thus, the level of anti-spike RBD IgG can be quantitatively visualized by the length of PMP accumulation without using any detection module.

Here, without relying on any detection module, we report a decentralized, instrument-free microfluidic device that allows direct visualization of SARS-CoV-2 antibody levels (Fig. 1 and movie S1). IgG antibodies against the SARS-CoV-2 spike protein receptor-binding domain (anti-spike RBD IgG) were chosen as the detection target because of their ability to neutralize both live and pseudotype SARS-CoV-2 viruses (*39*, *40*). To detect these antibodies, magnetic microparticles (MMPs) are modified with spike protein RBD to ensure the specific binding to anti-spike RBD IgG, while polystyrene microparticles (PMPs) are modified with secondary antibody against human IgG (anti-human IgG). Thus, the present anti-spike RBD IgG would simultaneously bind to MMPs and PMPs, forming MMPs-antibodies-PMPs. Loaded onto a capillary-driven microfluidic chip, the particle solution first flows through a magnetic separator that removes MMPs and MMPs-antibodies-PMPs. At the same time, free PMPs continue to flow until they are trapped at a particle dam. As a result, PMP accumulation is formed with a length inversely proportional to the antibody level and quantifiable by the naked eye. After validating the successful connection between MMPs and PMPs using a magnetophoresis assay, the analytical performance, i.e., limit of detection (LOD), selectivity, and comparison to commercial LFIA, was tested. Next, using undiluted serum, the platform was adapted to sensitive mode (LOD: 13.3 ng/ml; sample-to-answer time: 70 min; dynamic range: 5 to 1000 ng/ml) or rapid mode (LOD: 57.8 ng/ml; sample-to-answer time: 20 min; dynamic range: 50 to 3000 ng/ml). Last, 91 vaccinees were recruited to detect the plasma antibody levels using our microfluidic device and study the decay with time. By comparing the levels detected by the gold standard ELISA, high accuracy was achieved. This work demonstrates a microfluidic platform allowing low-cost, user-friendly interfaces, power-free operation, and quantification by visual inspection, making it particularly suitable for time-saving mass-screening settings or individual use in monitoring immune status after vaccination.

## RESULTS

### Magnetophoresis assay for validating particle connections

**Fig. 2.**
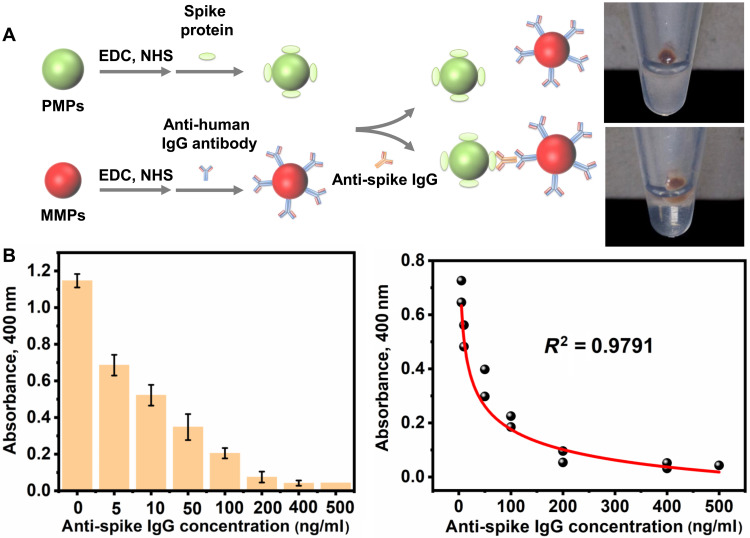
Magnetophoresis assay for validating particle connections. (**A**) Schematic illustration of the working principle of magnetophoresis assay. An external magnetic field pulls MMPs and PMPs-antibodies-MMPs to the sidewall of a test tube, leaving only PMPs freely suspended in the solution. Thus, the presence of antibodies is reflected by the turbidity of the supernatant. (**B**) Absorbance at 400-nm wavelength with serial dilution of anti-spike IgG in buffered solution (left) and nonlinear regression from 5 to 500 ng/ml (right) (mean ± SD, *n* = 2).

We first used a magnetophoresis assay (*41*) to validate the particle connection in response to the SARS-CoV-2 antibody (Fig. 2A). Anti-human IgG antibodies were immobilized on carboxyl-functionalized MMPs, while SARS-CoV-2 glycosylated spike protein was immobilized on carboxyl-functionalized PMPs. Hence, the particle system is specifically reactive to IgG antibodies against SARS-CoV-2 spike protein (anti-spike IgG). Here, the trimerized spike protein was chosen as the antigen because its binding to the angiotensin-converting enzyme 2 (ACE2) receptor is essential for cell entry and infection (*42*). Once anti-spike IgG is present, PMPs-antibodies-MMPs are formed. When an external magnetic field is applied, MMPs and PMPs-antibodies-MMPs are pulled to the sidewall of a test tube, leaving only PMPs freely suspended in the solution. Thus, the formation of PMPs-antibodies-MMPs would reduce the turbidity of supernatant, which can be observed visually or quantified by optical absorbance through an ultraviolet-visible (UV-Vis) spectrometer.

The microparticle system was first optimized using flow cytometry. The amount of anti-human IgG antibody on MMPs and SARS-CoV-2 spike protein on PMPs was optimized as 285 μg of anti-human IgG antibody per milligram of MMPs (0.797 μm in diameter) and 30 μg of spike protein per milligram of PMPs (0.955 μm in diameter) (fig. S1). On the basis of the optimized condition, the optical absorbance of supernatant after magnetic separation was measured, which showed a decrease of turbidity with an increased concentration of anti-spike IgG (Fig. 2B). Using nonlinear fitting, the regression equation was obtained as *y*_(5−500 ng/ml)_ = 0.8794 − 0.141ln(*x*) (*R*^2^ = 0.9791). Together, the reduced solution turbidity in response to the presence of anti-spike IgG validates the successful formation of PMPs-antibodies-MMPs.

### Visual detection via microfluidic particle accumulation

**Fig. 3.**
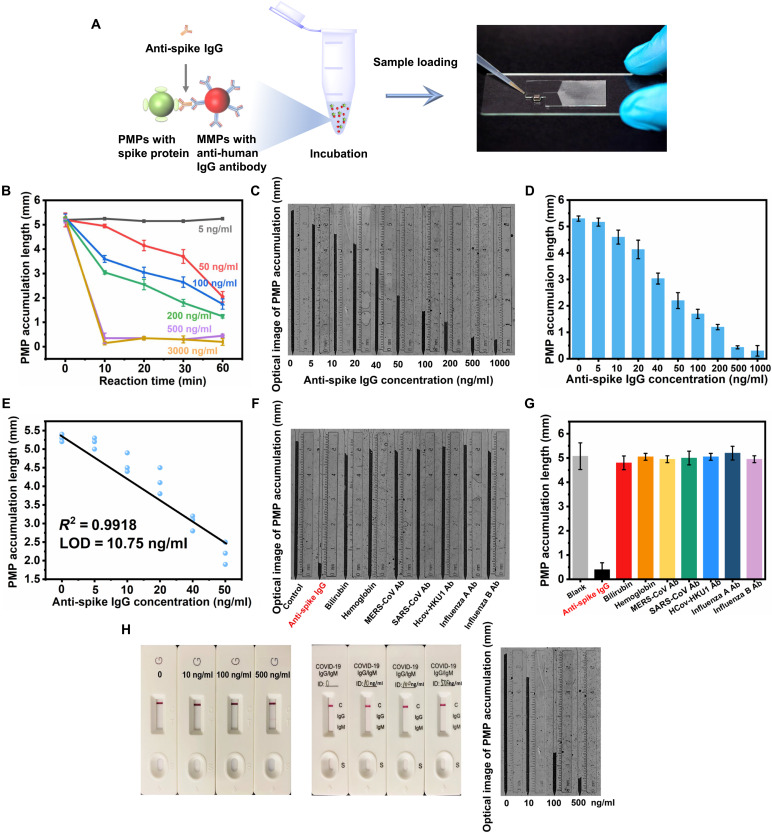
Visual quantification of anti-spike IgG by PMP accumulation in the microfluidic chip. (**A**) Schematic illustration of the operational steps. Coated MMPs and PMPs are incubated with anti-spike IgG in buffered solution for 1 hour before loading to the microfluidic chip. (**B**) Optimization of the incubation time for the formation of PMPs-antibodies-MMPs, which shows that 1-hour incubation is required to differentiate anti-spike IgG concentration lower than 50 ng/ml (mean ± SD, *n* = 3). (**C**) Optical image and (**D**) measured PMP accumulation length (mean ± SD, *n* = 3), which shows an inverse proportion to the target concentration. (**E**) Linear regression from 0 to 50 ng/ml. (**F**) Optical image and (**G**) measured PMP accumulation length showing that only anti-spike IgG (500 ng/ml) shortened the PMP accumulation, while other interfering substances and pathogen-specific antibodies with higher concentration (bilirubin, 125 μg/ml; hemoglobin, 4.5 mg/ml; MERS-CoV antibody, 1 mg/ml; SARS-CoV antibody, 1 mg/ml; HCoV-HKU1 antibody, 0.5 mg/ml; influenza A antibody, 1 mg/ml; influenza B antibody, 1 mg/ml) did not cause nonspecific binding between MMPs and PMPs (mean ± 95% confidence interval, *n* ≥ 2). (**H**) Comparison of an IgG antibody detection kit (Abcam, left) and an IgG/IgM antibody detection kit (Wuhan UNscience Biotechnology Co. Ltd., middle) to this work (right). While the color bands were only barely visible in anti-spike IgG (500 ng/ml) in one detection kit (Abcam, left), our device can already clearly differentiate the anti-spike IgG of 10 ng/ml.

While the presence of anti-spike IgG can be visually determined by the magnetophoresis assay, turbidity change is barely quantifiable by the naked eye. Hence, a simple and user-friendly microfluidic circuitry was developed to enable automated magnetic separation and quantification of altered particle connections through direct visual inspection. After the PMPs-antibodies-MMPs are formed, the microparticle solution is loaded into a capillary-driven microfluidic device containing a stomach-shape magnetic separator, a trapping channel, a particle dam with a narrowing nozzle, and a capillary pump region (Fig. 3A and fig. S2) (*43*). The microfluidic device was made by plasma bonding between a glass slide and a patterned Norland Optical Adhesive 63 (NOA 63) layer, so that stable hydrophilicity can be achieved (*44*, *45*). After loading, the solution first passed the magnetic separator that attracted the MMPs and PMPs-antibodies-MMPs to the side, leaving free PMPs to continue flowing until trapped at a particle dam. Thus, the presence of anti-spike IgG lessened the PMPs trapped at the particle dam (movie S2) compared to blank control (movie S3). To achieve the effect of the “dam,” the narrowest nozzle width was designed as 8 μm, which can effectively trap the PMPs (diameter: 15.34 μm). Furthermore, to better visualize the PMP accumulation length, the channel height in the NOA 63 layer was designed as 25 ± 0.3 μm, which allows PMPs to accumulate as monolayered in the trapping channel (movie S4) with a length inversely proportional to the antibody level and quantifiable by the naked eye.

The amount of MMPs and PMPs and the immobilized anti-human IgG antibody and SARS-CoV-2 spike protein were optimized for this microfluidic particle system. The PMP concentration was optimized to 20 mg/ml based on the highest signal-to-noise ratio (fig. S3A), and this PMP concentration was used to determine the amount of spike protein on PMPs (7.5 μg per milligram of PMPs) (fig. S3B). With optimized PMPs, the MMP concentration was serially diluted such that each MMP could receive more anti-spike IgG during particle connection. Using MMPs (5 mg/ml) at the optimized concentration (fig. S3C), 0.4 mg of anti-human IgG antibody per milligram of MMPs was found (fig. S3D).

Analytical performance was studied using anti-spike IgG diluted in buffered solution for reaction with optimized MMPs and PMPs. For concentrations of anti-spike IgG lower than 50 ng/ml, a 1-hour incubation is required (Fig. 3B). Next, LOD was then determined by serial dilution of anti-spike IgG (5, 10, 20, 40, 50, 100, 200, 500, and 1000 ng/ml). The increment of anti-spike IgG concentration would generate more MMPs-antibodies-PMPs, hence shortening the length of PMP accumulation in the channel (Fig. 3, C and D). On the basis of the linear range near the blank sample (0 to 50 ng/ml) (Fig. 3E), the linear regression was determined as $y_{\left(0-50\;\text{ng}/\text{ml}\right)}=5.3423-0.061x\pm 0.2892{\left(\frac{7}{18}+\frac{{\left(x-20.83\right)}^{2}}{4520}\right)}^{1/2}$ using least-squares regression while incorporating the variance of model parameters, i.e., uncertainties of intercept *s*_b_0__ and slope *s*_b_1__. A LOD of 10.75 ng/ml was achieved, which prevents type I and type II errors (less than 5% of false positive/negative rate) (see the Supplementary Materials for details) (*46*–*51*).

To explore the selectivity of this system, other potential interfering factors such as bilirubin, hemoglobin, and different pathogen-specific antibodies, i.e., Middle East respiratory syndrome coronavirus (MERS-CoV) antibody, SARS-CoV antibody, human coronavirus HKU1 (HCoV-HKU1) antibody, influenza A antibody, and influenza B antibody, were tested (Fig. 3, F and G). While a short PMP accumulation length (around 0.5 mm) was observed with anti-spike IgG (500 ng/ml), even high concentrations (~2000 times higher) of other interfering factors all resulted in long PMP accumulation lengths comparable to the blank control sample (*P* > 0.2 for all cases). Notably, on the basis of the 95% confidence interval of the two-tailed hypothesis (α = 0.025), the acceptable tolerance about blank control was calculated as 5.075 ± 0.55 mm, which covers the measured results from all interfering factors. Together, these findings suggest that the binding between MMPs and PMPs is specific to antibodies against SARS-CoV-2 spike protein.

This microfluidic chip was also compared to conventional rapid tests using the lateral flow method. For different concentrations (0, 10, 100, and 500 ng/ml) of anti-spike IgG, only the rapid test from Abcam shows a weak signal when the concentration is 500 ng/ml, and no sign for all the other cases (Fig. 3H, left). For another rapid test (Wuhan UNscience Biotechnology Co. Ltd), the results are negative for all concentrations of anti-spike IgG (Fig. 3H, middle). In contrast, PMP accumulation length in our device shows apparent differences compared to blank control even when anti-spike IgG concentration is as low as 10 ng/ml and continuously decreases for a higher concentration of anti-spike IgG (Fig. 3H, right). Together, the visual detection based on PMP accumulation provides clear-cut, quantitative results with substantially improved sensitivity and selectivity.

### Detection of anti-spike RBD IgG in undiluted serum

**Fig. 4.**
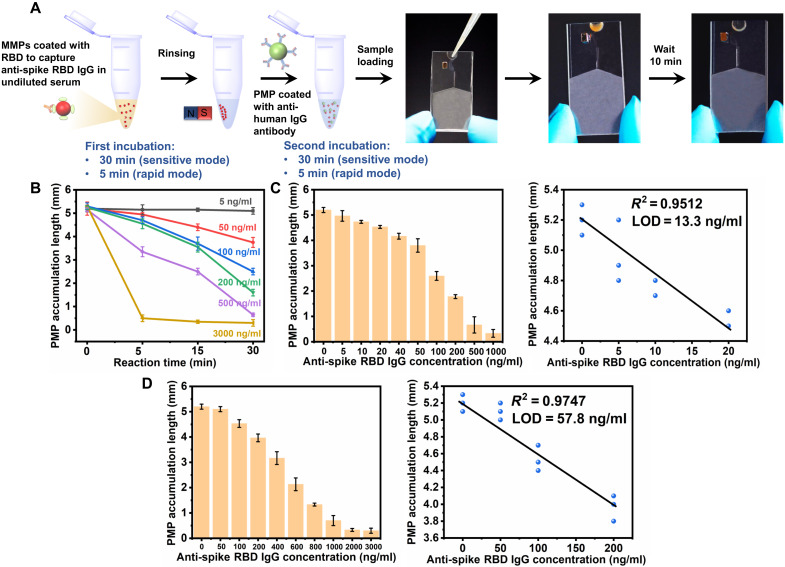
Detection of anti-spike RBD IgG in undiluted serum. (**A**) Schematic illustration of the operational steps for undiluted serum samples. MMPs coated with RBD are first mixed with serum to capture the anti-spike RBD IgG and incubated for 30 min (sensitive mode) or 5 min (rapid mode). After rinsing, PMPs coated with anti-human IgG antibodies are added and incubated for 30 min (sensitive mode) or 5 min (rapid mode) before loading to the PDMS loading chamber and placing the microfluidic chip vertically. Hence, the particle solution can enter the hydrophilic microchannel without additional handling. (**B**) Optimization of reaction time for both first and second incubation (mean ± SD, *n* = 3). (**C**) Measured PMP accumulation length with respect to varied concentrations of anti-spike RBD IgG in human serum (mean ± SD, *n* = 3) using sensitive mode (left) and the linear regression from 0 to 20 ng/ml (right). (**D**) Measured PMP accumulation length with respect to varied concentrations of anti-spike RBD IgG in human serum (mean ± SD, *n* = 3) using rapid mode (left) and the linear regression from 0 to 200 ng/ml (right).

After validating the successful detection of anti-spike IgG in the buffer, detection of undiluted serum samples was explored. We narrowed the detection target to anti-spike RBD IgG, a subtype of anti-spike IgG, because of its ability of virus neutralization (*39*, *40*). Notably, while other interfering substances may not cause nonspecific bindings between MMPs and PMPs (Fig. 3, F and G), they may still compete to bind microparticles coated with anti-human IgG antibodies when coexisting with the anti-spike RBD IgG. To specifically extract the anti-spike RBD IgG from such a complex environment, MMPs immobilized with SARS-CoV-2 glycosylated spike protein RBD were first mixed with undiluted serum (first incubation) to specifically capture the anti-spike RBD IgG (Fig. 4A, left). By rinsing the MMPs using a magnetic rack, the interfering materials such as other human antibodies can be removed before incubating with PMPs coated with anti-human IgG antibodies (second incubation). In this way, the detection can be operated through simple pipetting, making it an instrument-free and decentralized process.

On the other hand, in our previous design (Fig. 3A) (*44*, *45*), the chip was placed horizontally during capillary wicking. Because of gravitational sedimentation, most microparticles would settle without entering the microchannel during the entire process. Thus, resuspension and redeposition of microparticle solution are needed, which is labor intensive and experience dependent. In our new design, a polydimethylsiloxane (PDMS) loading chamber was designed and cast using a three-dimensional (3D)–printed mold. This design has two main features. First, because of the high capacity of the loading chamber, the device can be placed vertically with particle solution fully loaded (Fig. 4A, right). Hence, all microparticles will be introduced into the hydrophilic microchannel using capillary force with the assistance of gravitational sedimentation, making it easy to use without additional handling. Second, the inner surface of the PDMS loading chamber was coated with hydroxy-terminated PDMS as a lubricant. This coating, called the SLIP-LAB (Slippery Liquid-Infused Porous Surface Laboratory) concept, prepares the microchannel with excellent repellence to particle stiction (*52*). With this modification, the microfluidic device consists of PDMS as the bottom layer and NOA 63 as the top layer (fig. S4). After incubating the serum sample with rinsed MMPs and PMPs, the solution was dispensed into the loading chamber, and the device was placed vertically. Subsequently, the solution entered the microchannels in the NOA 63 layer and formed PMP accumulation in the trapping channel visible to the naked eye within 10 min. The operation procedure can be viewed by a demonstration video (movie S1).

To improve detection sensitivity, the amount of RBD immobilized on MMPs and anti-human IgG antibody coated on PMPs were optimized. By testing a series of the amount of RBD and anti-human IgG antibody, 20 μg of RBD per milligram of MMPs and 60 μg of anti-human IgG antibody per milligram of PMPs were selected (fig. S5). Additionally, the reaction time for both the first and second incubations was explored to make it more suitable for a rapid test. Based on the results of incubation time (Fig. 4B), two detection modes were designed. When 30 min was used for both the first and second incubations, a LOD of 13.3 ng/ml was achieved with a dynamic range from 5 to 1000 ng/ml [Fig. 4C; linear regression: $y_{\left(0-20\;\text{ng}/\text{ml}\right)}=5.1467-0.033x\pm 0.182{\left(\frac{5}{12}+\frac{{\left(x-8.75\right)}^{2}}{437.5}\right)}^{1/2}$]. This detection is named sensitive mode as it is suitable for serum samples with a lower concentration of anti-spike RBD IgG. In contrast, when both incubations were shortened to 5 min, a LOD of 57.8 ng/ml was achieved, with a dynamic range from 50 to 3000 ng/ml [Fig. 4D; linear regression: $y_{\left(0-200\;\text{ng}/\text{ml}\right)}=5.1533-0.0052x\pm 0.0281{\left(\frac{5}{12}+\frac{{\left(x-175\right)}^{2}}{1400}\right)}^{1/2}$]. The second mode is for rapid detection as it is particularly useful to rapidly determine the antibody concentration in serum with higher antibody levels.

We have also attempted the application of undiluted whole-blood samples. Using different concentrations of anti-spike RBD IgG diluted in whole human blood and 5 min for both incubations, the analytical performance was still achieved with a LOD of 1048.1 ng/ml (fig. S6). This LOD increases around 18 times higher than that of serum samples, which is consistent with our previous results of detection of lead intoxication in human serum and whole blood (*45*) and possibly due to the adsorption of hemocytes or unwanted cellular debris on microparticles. Thus, given the inherent complexities and potential issues of coagulation, blood serum/plasma is still preferred, which can be obtained by other microfluidic devices (*53*) or hand-powered centrifuge (*54*) in resource-limited settings.

### Detection of anti-spike RBD IgG in COVID-19 vaccinees

**Fig. 5.**
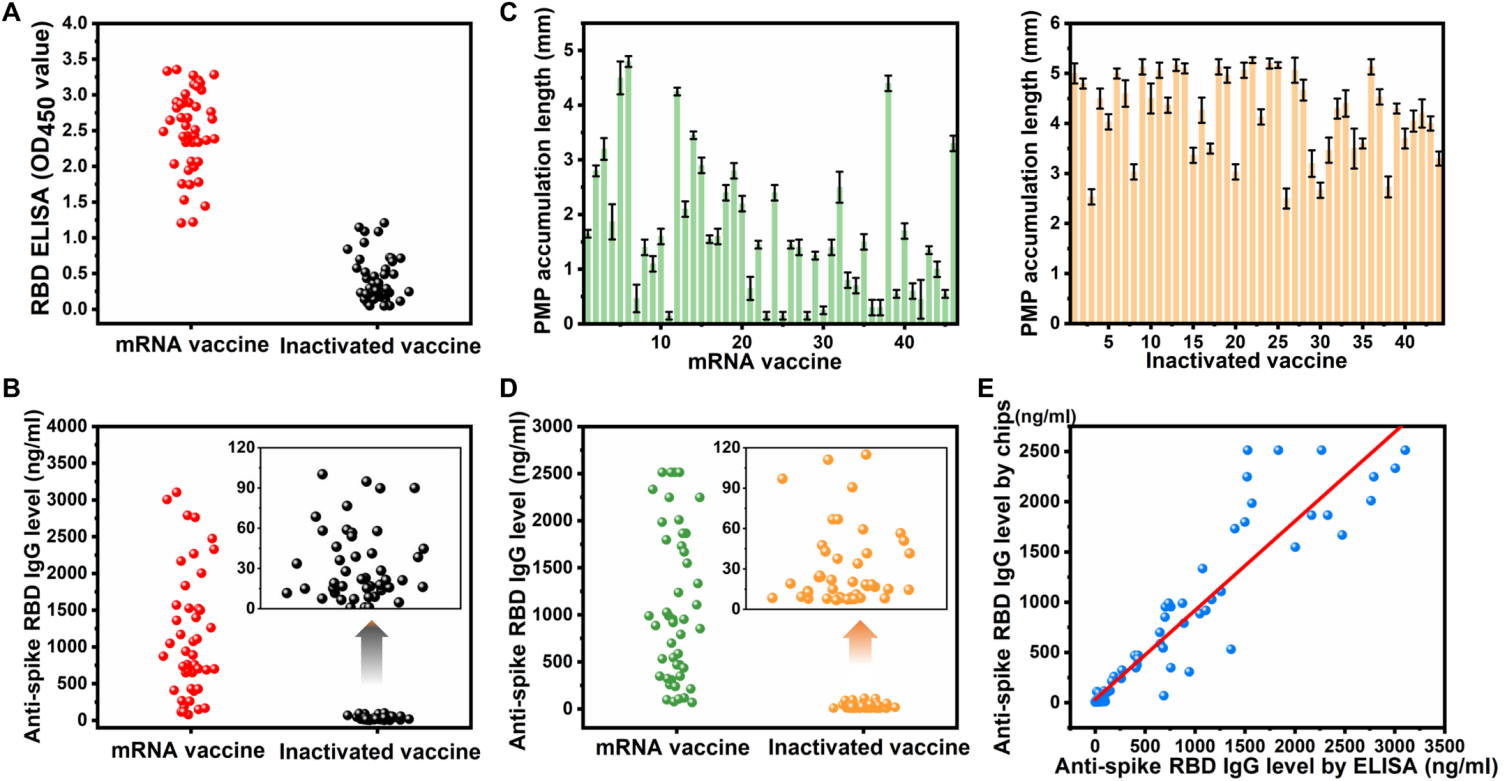
Level of anti-spike RBD IgG in vaccinees of mRNA and inactivated vaccine. (**A**) OD_450_ values of 91 volunteers (46 for the mRNA vaccine and 45 for the inactivated vaccine) were tested by an anti-spike RBD IgG ELISA kit. (**B**) Calculated anti-spike RBD IgG levels based on ELISA showing the mean (range) as 1124.8 (100 to 3200) ng/ml for the mRNA vaccine and 32.5 (0 to 100 ng/ml) for the inactivated vaccine. (**C**) PMP accumulation length by microfluidic chips for 46 mRNA vaccinees using rapid mode (left) and 45 inactivated vaccinees using sensitive mode (right) (mean ± SD, *n* = 3). (**D**) Calculated anti-spike RBD IgG levels based on microfluidic chip test [1080.8 (100 to 2500) ng/ml for mRNA vaccine and 30.2 (0 to 120) ng/ml for inactivated vaccine]. (**E**) Comparison of anti-spike RBD IgG levels measured by an anti-spike RBD IgG ELISA kit (*x* axis) and microfluidic chips (*y* axis). The accuracy was determined on the basis of Lin’s concordance correlation coefficient ${\hat{\rho }}_{c}$= 0.935, validating good agreement between our microfluidic chip and the gold standard ELISA.

The above experimental results show that our method has excellent analytical performance for detection in undiluted serum, suggesting great potential for detecting antibody levels in human plasma of COVID-19 vaccinees. Ninety-one volunteers with no prior history of COVID-19 before receiving COVID-19 vaccines were recruited in Hong Kong. All volunteers have received two doses of vaccines. Of the volunteers, 51 were women, and 40 were men. Forty-six vaccinees (23 males) received mRNA vaccines (BNT162b2, BioNTech), and 45 vaccinees (17 males) received inactivated vaccines (Coronavac, Sinovac). The mean (range) age was 26 (18 to 60) years old, and 85% were 20 to 40 years old. There were no significant differences in mean (range) age between mRNA and inactivated vacccines [25.1 (18 to 60) versus 27.4 (19 to 39); *P* = 0.086]. Finger-prick blood was collected from each volunteer participant. The mean (range) interval (days) between the second dose of vaccine and blood collection is 50.08 (4 to 141) [mRNA vaccinees: 41.4 (4 to 86); inactivated vaccinees: 60.7 (6 to 141); *P* = 0.0016]. Absolute ELISA first measured the plasma samples of 91 volunteers. A standard curve was created using serial dilutions of anti-spike RBD IgG in human serum (fig. S7). As seen by optical density at the wavelength of 450 nm (OD_450_) (Fig. 5A), the level of IgG antibody against RBD arising from the mRNA vaccine is much higher than that from the inactivated vaccine. Using inverse regression referencing to the calibration curve (fig. S7), the mean (range) level of anti-spike RBD IgG can be determined as 1124.8 (100 to 3200) ng/ml for mRNA vaccine, which is significantly greater than the 32.5 (0 to 100 ng/ml) for the inactivated vaccine (*P* < 0.001) (Fig. 5B).

The interval between the second dose of vaccine and the first blood collection in inactivated vaccinees is significantly longer than that in mRNA vaccinees (*P* = 0.0016), which may partly account for the differences in anti-spike RBD IgG levels. Hence, the antibody level of vaccinees within the range from days 21 to 35 after the second dose of vaccination was further studied. Within the selected range, there were no significant differences between two vaccine groups in the interval (days) from the second dose of vaccination to the day of measurement [mRNA vaccinees (*n* = 4, 29.7 days) versus inactivated vaccinees (*n* = 10, 29.25 days); *P =* 0.845]. Significant differences in mean (range) level of anti-spike RBD IgG in mRNA vaccinees and inactivated vaccinees [1109.2 (105.9 to 2513.3) ng/ml versus 62.1 (33. 9 to 114.9) ng/ml; *P* = 0.014] were still found. Thus, the mRNA vaccine leads to much greater immunogenicity than does the inactivated vaccine.

On the basis of the ELISA results and the optimized experimental conditions, different modes were adopted for testing on microchips, i.e., the rapid mode for mRNA vaccinees and the sensitive mode for inactivated vaccinees. After measuring the PMP accumulation length (Fig. 5C), the anti-spike RBD IgG levels of 91 volunteers were calculated according to the regression equations (Fig. 4, C and D, and fig. S8). The mean (range) level of anti-spike RBD IgG is significantly higher in mRNA vaccinees than in inactivated vaccinees [1080.8 (100 to 2500) ng/ml for mRNA vaccinees versus 30.2 (0 to 120) ng/ml for inactivated vaccinees; *P* < 0.001] (Fig. 5D) and very similar to the results of ELISA. The microfluidic test results were compared to the ELISA test results on a one-to-one basis to validate the accuracy (Fig. 5E). Notably, better agreement is observed when concentration is low, because the response becomes nonlinear when concentrations go high in both calibration curves (figs. S7 and S8). Thus, a slight variance in measured signals (PMP accumulation length or OD_450_ value) causes considerable fluctuation of calculated antibody concentration. Therefore, the results from samples with low concentration are more comparable than those with high concentrations. Overall, a good correlation was observed on the basis of Lin’s concordance correlation coefficient (${\hat{\rho }}_{c}$ = 0.935) (see the Supplementary Materials for details) (*55*), suggesting that the microfluidic chip test is a reliable device for quantification of anti-spike RBD IgG antibodies comparable to the conventional gold standard ELISA.

Ninety-one volunteers were also tested by LFIA for comparison. For mRNA and inactivated vaccines, the sensitivity is 6.52 and 46.67%, respectively (fig. S9 and table S1), suggesting low sensitivity of conventional LFIA. Moreover, unlike LFIA, which would give ambiguous results as shown by traditional color bands, our microfluidic particle dam can provide rapid quantification by the growth of a visual bar composed of PMP accumulation. Similar in principle to a mercury thermometer, this device is clear cut, accurate, and convenient to end users without the need for specific readers.

### Comparison of age, gender, days after vaccination, and decay in antibody level

**Fig. 6.**
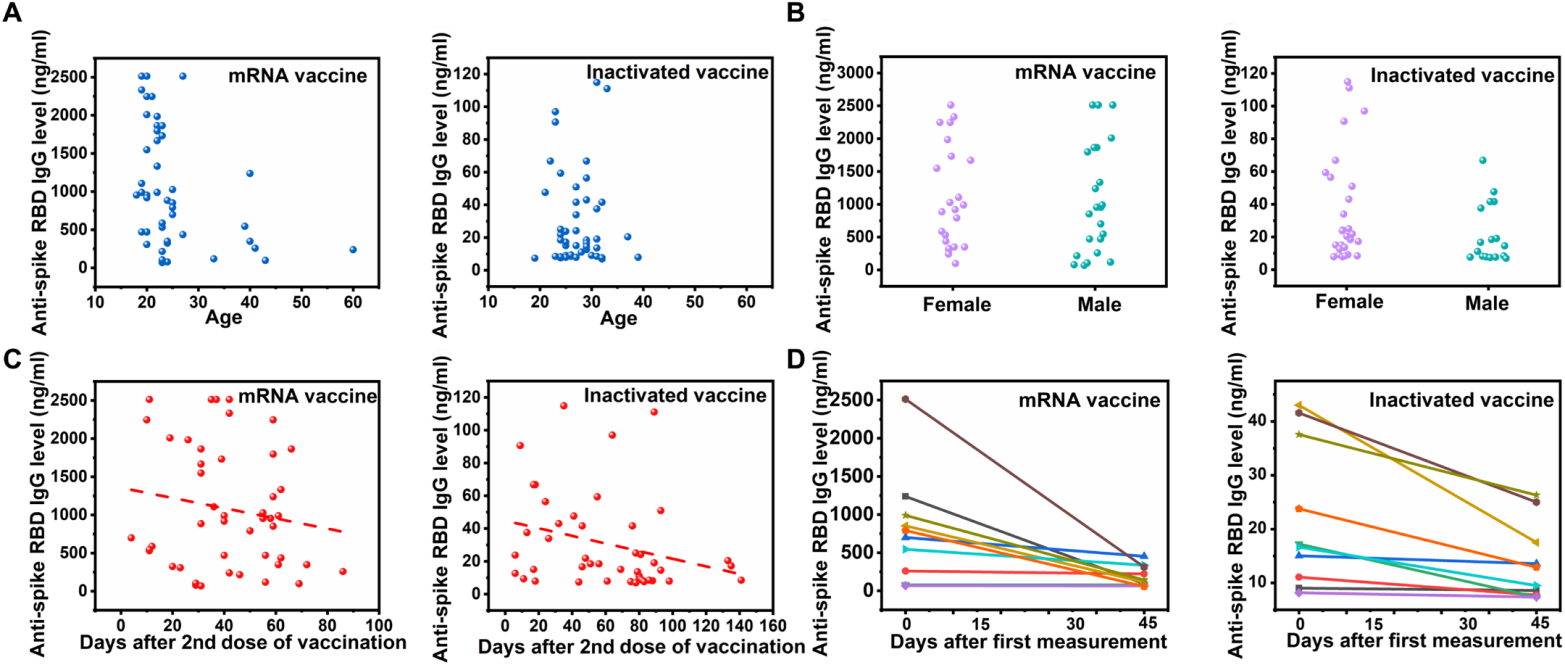
Level of anti-spike RBD IgG based on age, gender, and time by microfluidic chip. (**A** to **C**) Level of plasma anti-spike RBD IgG based on age (A), gender (B), and days from the second dose of vaccine (C). The dashed line in (C) is the linear regression. (**D**) Anti-spike RBD IgG level of 20 vaccinees of the first measurement and on the 45th days after the first measurement.

With the success of antibody detection, we next explored whether the age and gender would affect antibody levels in different groups of vaccinees. For mRNA vaccine, the anti-spike RBD IgG level is significantly higher in volunteers at the age of 18 to 22 compared to the others (23 to 60; *P* < 0.001) and independent to the gender (*P* = 0.835) (Fig. 6, A and B, and fig. S10, A and B). For inactivated vaccines, the anti-spike RBD IgG level is generally low regardless of the age or gender. In addition, the decay of anti-spike RBD IgG level with time was also evaluated. Generally, the anti-spike RBD IgG level decreases with time after the second dose for both vaccine types, as seen by the negative slope of linear regressions (Fig. 6C and fig. S10C).

To further elucidate the decay in antibody level, a second measurement of antibody level of 20 volunteers (10 mRNA vaccinees and 10 inactivated vaccinees) was repeated on the 45th day after the first measurement (Fig. 6D and fig. S10D). The anti-spike RBD IgG level in each mRNA vaccinee decays significantly on the 45th day (*P =* 0. 0179). For inactivated vaccinees, the already low anti-spike RBD IgG level at first measurement further decreased on the 45th day (*P =* 0.007). Together, these results demonstrate a decay in anti-spike RBD IgG levels among vaccinees, suggesting the need to evaluate antibody levels as surrogates of immune protection.

## DISCUSSION

Increasing evidence suggests SARS-CoV-2 antibody level as a correlate to immune protection, which is useful when choosing vaccine types and monitoring its decay over time. Antibody-based “immunity passports,” instead of vaccination records alone, might offer a better additional means of evaluation before resuming work, flight travel, or border entry control (*56*). In this work, we present a decentralized, instrument-free microfluidic device for direct visualization of SARS-CoV-2 antibody levels in the COVID-19 vaccinees. The LOD and dynamic range can be tuned by adjusting the incubation time to suit different scenarios, i.e., for higher sensitivity (LOD: 13.3 ng/ml; sample-to-answer time: 70 min; dynamic range: 5 to 1000 ng/ml) or rapid tests (LOD: 57.8 ng/ml; sample-to-answer time: 20 min; dynamic range: 50 to 3000 ng/ml). By testing undiluted plasma samples from vaccinees, the mean (range) of anti-spike RBD IgG level is 1080.8 (100 to 2500) ng/ml for mRNA vaccinees and 30.2 (0 to 120) ng/ml for inactivated vaccinees, and both levels decay significantly in 45 days. Compared to conventional lateral flow strips, our microfluidic device is equally simple to use, instrument-free, and low cost but with greatly improved sensitivity and clear-cut, quantitative results by PMP accumulation. Similar to an ordinary mercury thermometer, the result is readable and unambiguous. Thus, our device is particularly suitable for the general public to routinely check immune protection at local clinics, testing booths at border entry control, or other public sectors, providing a solution for accelerating economic recovery without adding to the medical burden on health care systems.

## MATERIALS AND METHODS

### Reagents

2-(N-morpholino)ethanesulfonic acid (MES) was purchased from J&K Scientific. 1-Ethyl-3-(3-dimethylaminopropyl) carbodiimide hydrochloride (EDC), *N*-hydroxysuccinimide (NHS), powder of Dulbecco’s phosphate-buffered saline (PBS), 50% hydroxylamine, goat anti-human IgG antibody–Alexa Fluor 647 (catalog no. A21445), and normal human serum (catalog no. 31876) were obtained from Thermo Fisher Scientific. Single-donor human whole blood was purchased from Innovative Research. Bovine serum albumin (BSA) was acquired from Sigma-Aldrich, USA. The 50% Tween 20 solution was from Life Technologies, USA. Carboxyl MMPs (catalog no. MFY0002, 0.797 μm in diameter, 1.725 × 10^11^ microspheres/ml) and carboxyl PMPs (catalog no. PC04001, 0.955 μm in diameter, 2.07 × 10^10^ microspheres/ml and catalog no. PC07002, 15.3 μm in diameter, 5.146 × 10^7^ microspheres/ml) were purchased from Bangs Laboratories Inc., USA. Trimerized SARS-CoV-2 spike protein (catalog no. SPN-C82E3), SARS-CoV-2 spike protein RBD (mFc Tag) (catalog no. ABIN6961175), and anti–SARS-CoV-2 spike S1 (RBD) antibody (catalog no. ABIN6952639) were purchased from antibodies-online. Anti–SARS-CoV-2 spike S1 (RBD) antibody was used as the model target representing both anti-spike IgG and anti-spike RBD IgG depending on the detection settings of MMPs and PMPs. The SARS-CoV-2 IgG ELISA Kit (catalog no. ab275300) and the Novel Coronavirus IgG Antibody Detection Kit (catalog no. ab272243) were from Abcam. COVID-19 Rapid Test Kit IgG+IgM (catalog no. A122152) was purchased from Wuhan UNscience Biotechnology Co. Ltd. The disposable sterile blood collection needle was from Icare Medical Supply Inc., and the glass capillary with 75-μl capacity was from Hirschmann.

### Microparticle activation

The carboxylated MMPs and PMPs were activated by EDC and NHS. Twenty microliters of microparticle stock solution was washed two times with 200 μl of activation buffer (0.1 M MES in deionized water, pH 5.0). One hundred microliters of EDC (50 mg/ml) and 100 μl of NHS (50 mg/ml) freshly prepared in activation buffer were added to microparticle suspension to react 15 min at room temperature with continuous mixing. Then, the microparticles were washed three times by coupling buffer (0.2% Tween 20 in 0.01 M PBS, pH 8.5) and resuspended in 20 μl of coupling buffer.

### Flow cytometry

Flow cytometry was used to optimize the amount of anti-human IgG antibody on MMPs and trimerized SARS-CoV-2 spike protein on PMPs. For MMPs, 3.5 μl of activated MMPs (1.725 × 10^11^ microspheres/ml) and 2 μl of anti-human IgG antibody with different concentrations (fig. S1) were gently mixed at 4°C in coupling buffer overnight. The next day, they were washed three times by reaction buffer (1% Tween 20 in 0.01 M PBS, pH 8.5) and the volume was adjusted to 500 μl for flow cytometry test. For PMPs, 3.4 μl of activated PMPs (0.955 μm in diameter, 2.07 × 10^10^ microspheres/ml) and 2 μl of trimerized SARS-CoV-2 spike protein with different concentrations (fig. S1) were gently mixed in coupling buffer at 4°C overnight. The next day, they were washed and resuspended in 200 μl of blocking buffer (40 mM hydroxylamine, 1% BSA, and 1% Tween 20 in 0.01 M PBS buffer) for 30 min before washing three times with reaction buffer. Subsequently, 2 μl (2 μg/ml) of anti-spike RBD IgG spiked in reaction buffer was gently mixed for 1 hour at room temperature and washed three times to remove extra antibodies. Then, 2 μl (2 mg/ml) of anti-human IgG antibody was added and mixed for 1 hour at room temperature and washed three times with reaction buffer. Last, the PMPs with surface conjugates were washed three times and the volume was adjusted to 500 μl with reaction buffer for the flow cytometry test. Emission was collected by the allophycocyanin (APC) fluorescence channel, and 10,000 particles were analyzed for each sample. The average value was calculated to represent the fluorescence intensity.

### Magnetophoresis assay

For the magnetophoresis assay, the diameters of MMPs and PMPs are 0.797 and 0.955 μm, respectively. MMPs were first diluted 5× from stock (3.45 × 10^10^ microspheres/ml), while PMPs were diluted 10× from stock (2.07 × 10^9^ microspheres/ml). After activation described above, 3.5 μl of MMPs (3.45 × 10^10^ microspheres/ml) was mixed with 2 μl of anti-human IgG antibody, while 3.5 μl of PMPs (2.07 × 10^9^ microspheres/ml) was mixed with 2 μl of trimerized SARS-CoV-2 spike protein. The concentration of immobilized protein is based on the optimized results from flow cytometry (fig. S1). After incubation at 4°C overnight with constant mixing, the modified microparticles were washed and blocked in 200 μl of blocking buffer (40 mM hydroxylamine, 1% BSA, and 1% Tween 20 in 0.01 M PBS buffer) for 30 min with shaking. Subsequently, they were washed three times and resuspended in 3.5 μl of reaction buffer. Then, 3.5 μl of MMPs and 3.5 μl of PMPs were mixed with 2 μl of anti-spike IgG and 11 μl of reaction buffer followed by shaking for 1 hour for the connection between MMPs and PMPs. After that, the solution was placed onto a magnet rack for 2 min and observed by the naked eye or measured by optical absorbance using an UV-Vis spectrometer (BioDrop μLITE, UK).

### Design and fabrication of microfluidic chip

The device uses PDMS or glass slide as the bottom layer and NOA 63 as the top layer. To facilitate solution wicking on the glass slide, the outer region of the inlet was made hydrophobic by coating with trichloro (1*H*,1*H*,2*H*,2*H*-perfluorooctyl) silane (97%) (J&K Scientific Ltd.) diluted in toluene (10 weight percent) through gas-phase deposition inside a vacuum chamber for 40 min. Next, the slide was treated with plasma (Harrick Plasma; 800 mtorr, 2 min), with a tape covering the outer region of the sample loading inlet to protect the silane-treated glass surface. On the other hand, the PDMS layer embedded with a loading chamber of 2 mm in height was molded on the basis of a 3D-printed master (fig. S4). Briefly, the chamber was predesigned using AutoCAD software and exported as the STL file for 3D printing (Anycubic Photon Mono SE). The printed mold was first cleaned with 95% ethanol with sonication for 3 min as the user guide recommended. After that, the mold was treated with UV post-curing for 10 min and heated at 120°C for 2 hours to avoid PDMS curing inhibition issue (step 1 in fig. S4) (*57*). Next, the PDMS precursor (elastomer base: curing agent = 10:1; Sylgard TM184, Dow Corning, USA) was poured onto the 3D-printed mold, cured at 80°C for 2 hours (step 2 in fig. S4), peeled off, cut, and immersed in a hydroxy-terminated PDMS bath (viscosity: 25 cSt; Sigma-Aldrich, 481939) at 55°C overnight before drying it using an air gun (step 3 in fig. S4) (*52*). The NOA 63 bottom layer fabrication was based on traditional soft lithography, as previously mentioned (*44*). SU8 2015 photoresist (Gersteltec Sarl, Switzerland) was first spin-coated onto the silicon wafer (Suzhou Crystal Silicon Electronic & Technology Co. Ltd.) at 1200 rpm so that SU8 master with a depth of 25 ± 0.3 μm was obtained. After UV exposure and development, PDMS precursor was poured onto the SU8 master mold, cured at 80°C for 2 hours, and peeled off. Next, the patterned surface of the PDMS was treated with plasma (Harrick Plasma, 400 mtorr, 2 min) before being coated with trichloro (1*H*,1*H*,2*H*,2*H*-perfluorooctyl) silane (97%) (J&K Scientific Ltd.) by gas-phase deposition at room temperature for overnight, followed by a secondary PDMS casting on top of it. After curing and demolding, the secondary PDMS was used as the master (step 4 in fig. S4) where NOA 63 was smeared onto the patterned surface of the secondary PDMS and covered by a commercially available polypropylene film with 100 μm thickness (KOKUYO, Japan) before UV curing for 50 s (step 5 in fig. S4). After curing and peeling off (step 6 in fig. S4), NOA 63 with microchannel pattern was cut and bonded with the glass slide or the PDMS layer after plasma treatment (800 mtorr, 2 min) (step 7 in fig. S4). Then, a neodymium magnet in the size of 2.6 mm by 1.8 mm by 1.5 mm was glued at a 1-mm distance next to the magnetic separator.

### Detection in microfluidic chip

For the microfluidic particle system, the diameters of MMPs and PMPs are 0.797 and 15.3 μm, respectively. Before activation, the microparticle solution was diluted to the optimized concentration (fig. S3, A and C). Five microliters of diluted MMPs was mixed with anti-human IgG antibodies or anti-spike RBD IgG, while 5 μl of diluted PMPs was mixed with trimerized SARS-CoV-2 spike protein or anti-human IgG antibodies. The concentration of immobilized protein is based on the optimized results (figs. S3, B and D, and S5). After incubation at 4°C overnight with constant mixing, the modified microparticles were washed and blocked in 200 μl of blocking buffer for 30 min. For detection in buffered solution, the blocking buffer is 40 mM hydroxylamine, 1% BSA, and 1% Tween 20 in 0.01 M PBS buffer; for detection in undiluted serum/plasma, the blocking buffer was changed to 10% casein in PBS buffer. After blocking, the modified PMPs and MMPs were washed three times and resuspended in 5 μl of reaction buffer. After that, 5 μl of PMPs, 5 μl of MMPs, and 5 μl of anti-spike IgG or anti-spike RBD IgG spiked in the reaction buffer with different concentrations (total volume 15 μl) were gently mixed for 1 hour at room temperature. Last, 3 μl of particle solution from the 15 μl of total volume was loaded into the microfluidic chips. On the other hand, for detection in human serum/plasma or whole blood, 5 μl of MMPs was mixed with 5 μl of serum/plasma/blood samples and gently shaken for 30 or 5 min. After that, MMPs were washed three times with reaction buffer before bringing the volume to 10 μl, followed by adding 5 μl of PMPs and gentle shaking for 30 or 5 min. Last, 3 μl of particle solution from the 15 μl of total volume was loaded into microfluidic chips to test the PMP accumulation length.

### Enzyme-linked immunosorbent assay

The standard sample was created by serial dilutions of anti-spike RBD IgG in human serum. For ELISA test, standard sample and blank control in human serum, as well as volunteer plasma samples, were diluted 100 times by dilution solution before use. Next, 100 μl of diluted samples was added to the appropriate wells, followed by carefully covering the wells with a new sealing tape and incubating for 30 min at room temperature. Then, the adhesive plate cover was carefully removed, and the wells were washed five times with 250 μl of TNT (tris, NaCl, and Tween 20) wash buffer, followed by adding 100 μl of detection antibody to each well. After gently tapping the plate several times for mixing, a new adhesive plate cover was attached for incubation for 30 min at room temperature. After that, the adhesive plate cover was removed, and the wells were washed five times with TNT wash buffer. As follows, 100 μl of trimethylboron substrate solution was added into each well and incubated at room temperature in the dark for 15 min before adding 100 μl of stop solution to each well, tapping the plate gently to mix. The color of the solution in the wells immediately changed from blue to yellow. Using a microplate reader (SpectraMax M5e Multi-Mode Microplate Reader, Molecular Devices), OD_450_ was measured on the basis of the optical absorbance at 450 nm subtracted by the optical absorbance of 570 nm as the background noise within 30 min after the reaction stopped.

### Tests of volunteer specimens

Ninety-one volunteers vaccinated with COVID-19 vaccines in Hong Kong were recruited. Informed consent from each volunteer was obtained. The protocol was approved by the Human Subjects Ethics Subcommittee in City University of Hong Kong (reference no. 2-2021-41-F). Volunteers’ fingertips were disinfected with alcohol swabs. After alcohol evaporation, a disposable sterile blood collection needle was used to puncture the fingertips to collect the finger-prick blood using a glass capillary coated with an anticoagulant. The capillary drew 75 μl of blood. Last, volunteers’ fingers were wiped with a cotton swab moistened with alcohol and wrapped with an adhesive bandage. Blood collected by capillary blood collection device was transferred into a 0.5-ml anticoagulation tube. Then, 20 μl of whole blood was loaded into a lateral flow assay (COVID-19 Rapid Test Kit IgG+IgM from Wuhan UNscience Biotechnology Co. Ltd.) followed by three drops of buffer immediately. After 15 min, the band can be read and imaged. The remaining whole blood was centrifuged at 4°C, 10,500 rpm for 10 min to remove blood cells and isolate the plasma. The plasma was tested by ELISA and microfluidic chips as mentioned above.

### Statistical analysis

Descriptive statistics were used. Because of the skewed distribution of anti-spike RBD IgG levels, the Mann-Whitney *U* test was used to compare two groups using IBM SPSS Statistics version 28.0. For other data, the Student’s *t* test was used to compare two groups using the T.TEST function in Microsoft Excel. The paired *t* test was used to compare the first and second measurements (45th day after the first measurement) of the same subject, and an unpaired *t* test was used for other comparisons. A *P* value of <0.05 was considered statistically significant.

## Supplementary Materials

### This PDF file includes:

Figs. S1 to S10 Tables S1 and S2 Data processing and analysis

## Notes

View/request a protocol for this paper from *Bio-protocol*.
