# Supplementary material for: Microfluidic particle dam for direct visualization of SARS-CoV-2 antibody levels in COVID-19 vaccinees
Source: Sci Adv. 2022 Jun 3;8(22):eabn6064. doi: 10.1126/sciadv.abn6064 (PMC9166397; doi:10.1126/sciadv.abn6064)
Supplement: Supplementary file 1 — Figs. S1 to S10 Tables S1 and S2 Data processing and analysis [file sciadv.abn6064_sm.pdf]

Science Advances
AAAS

Supplementary Materials for

Microfluidic particle dam for direct visualization of SARS-CoV-2 antibody levels in COVID-19 vaccinees

Minghui Wu *et al.*

Corresponding author: Ting-Hsuan Chen, thchen@cityu.edu.hk

Sci. Adv. **8**, eabn6064 (2022)
DOI: 10.1126/sciadv.abn6064

The PDF file includes:

Figs. S1 to S10
Tables S1 and S2
Legends for movies S1 to S4
Data processing and analysis

Other Supplementary Material for this manuscript includes the following:

Movies S1 to S4

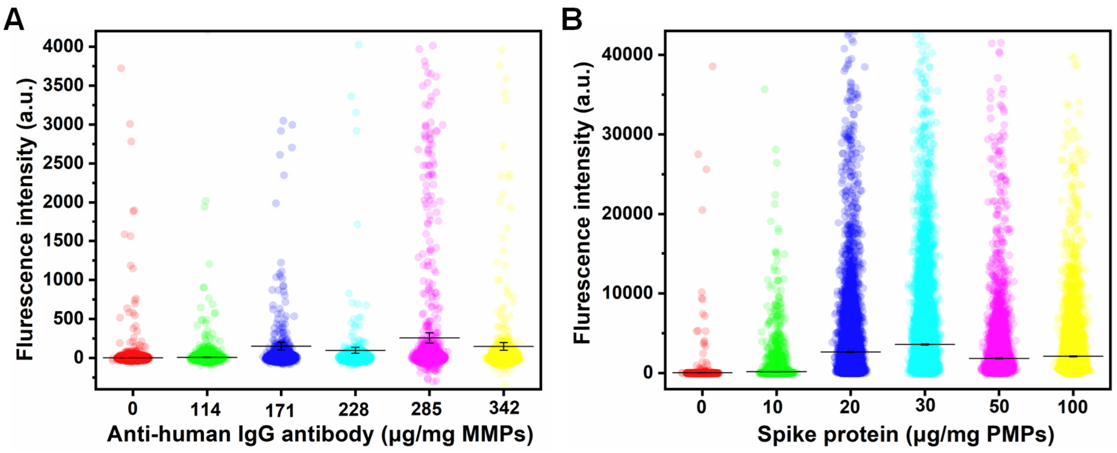

Fig. S1. Flow cytometry for optimization of protein immobilization of microparticles. The fluorescence intensity of (**A**) MMPs with a varied amount of anti-human IgG antibody and (**B**) PMPs with a varied amount of spike protein (mean ± SD, n = 10,000).

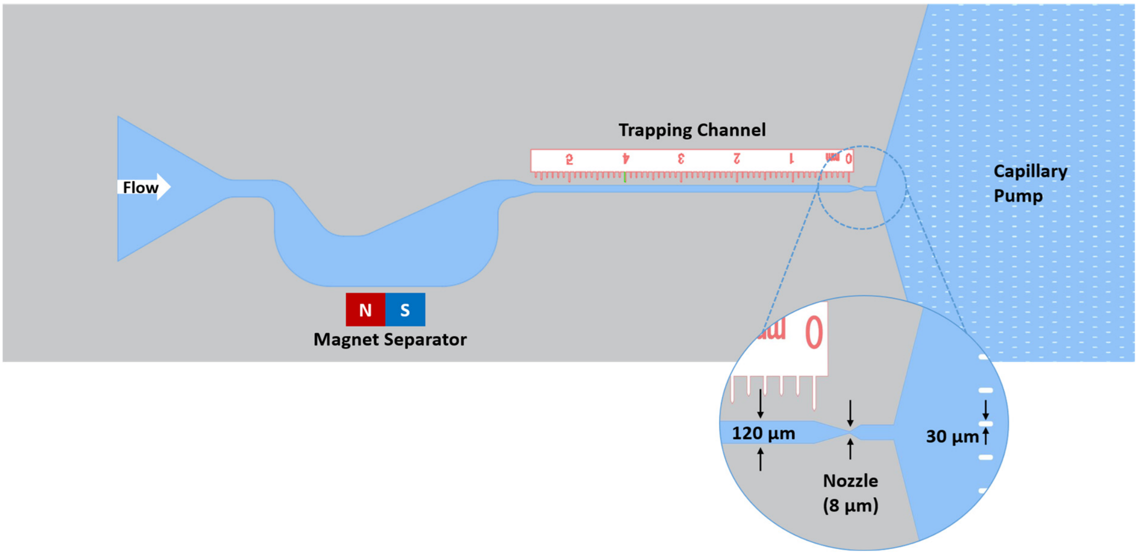

Fig. S2. The layout of the microfluidic channels. After passing the magnetic separator, the PMPs (diameter of 15.34 µm) are trapped at the particle dam with the narrowest nozzle width of 8 µm. Because the channel height is 25 ± 0.3 µm, PMPs would accumulate as a monolayer in the trapping channel with a length inversely proportional to the amount of anti-spike IgG or anti-spike RBD IgG and quantifiable by the naked eye.

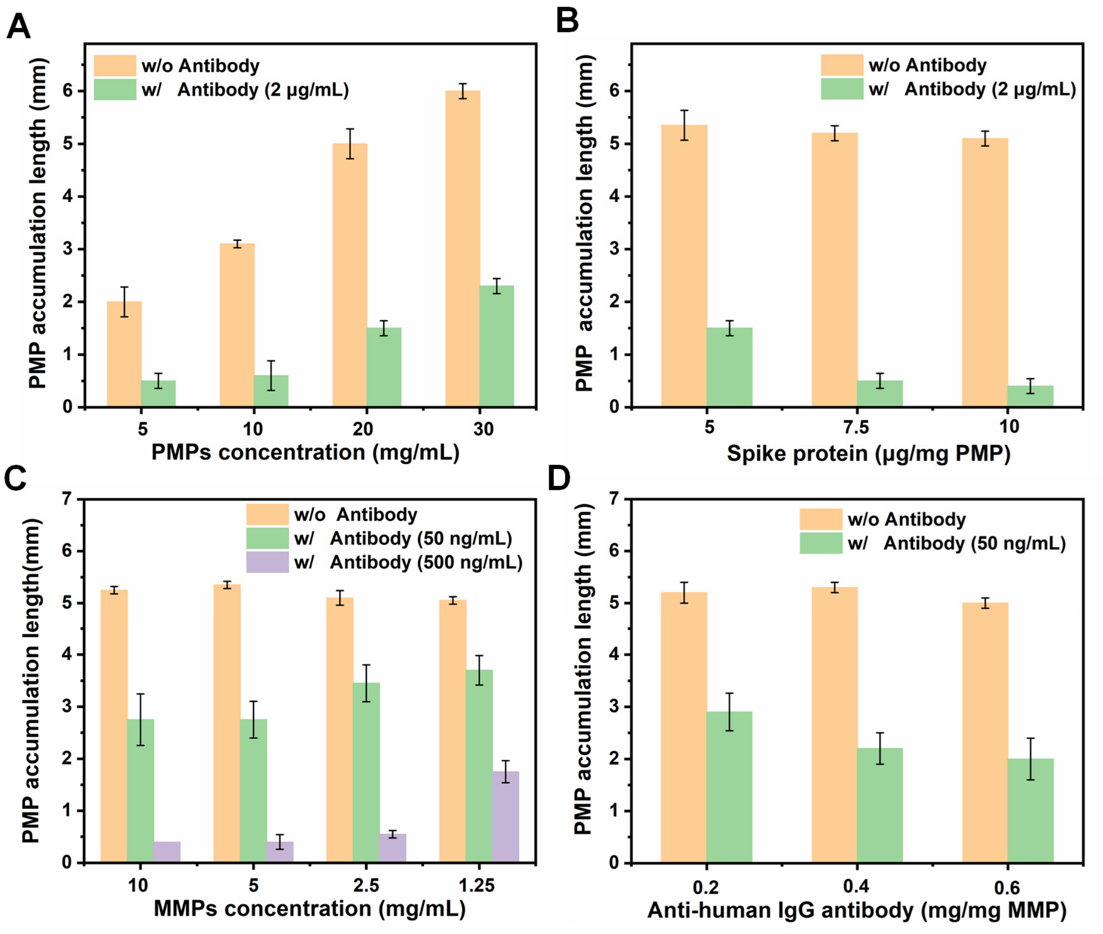

Fig. S3. Optimization of microparticle concentration and protein immobilization for detection of anti-spike IgG using microfluidic chips. The PMP accumulation length using varied (**A**) PMPs concentration (10 mg/ml of MMPs, 10 µg of spike protein per milligram of PMPs, 285 µg of anti-human IgG antibody per milligram of MMPs), (**B**) spike protein on PMPs (20 mg/ml of PMPs, 10 mg/mL of MMPs, 285 µg of anti-human IgG antibody per milligram MMPs), (**C**) MMPs concentration (20 mg/ml of PMPs, 7.5 µg of spike protein per milligram of PMPs, 285 µg of anti-human IgG antibody per milligram of MMPs), and (**D**) anti-human IgG antibody on MMPs (20 mg/ml PMPs, 5 mg/mL MMPs, 7.5 µg of spike protein per milligram of PMPs) (mean ± SD, n = 3).

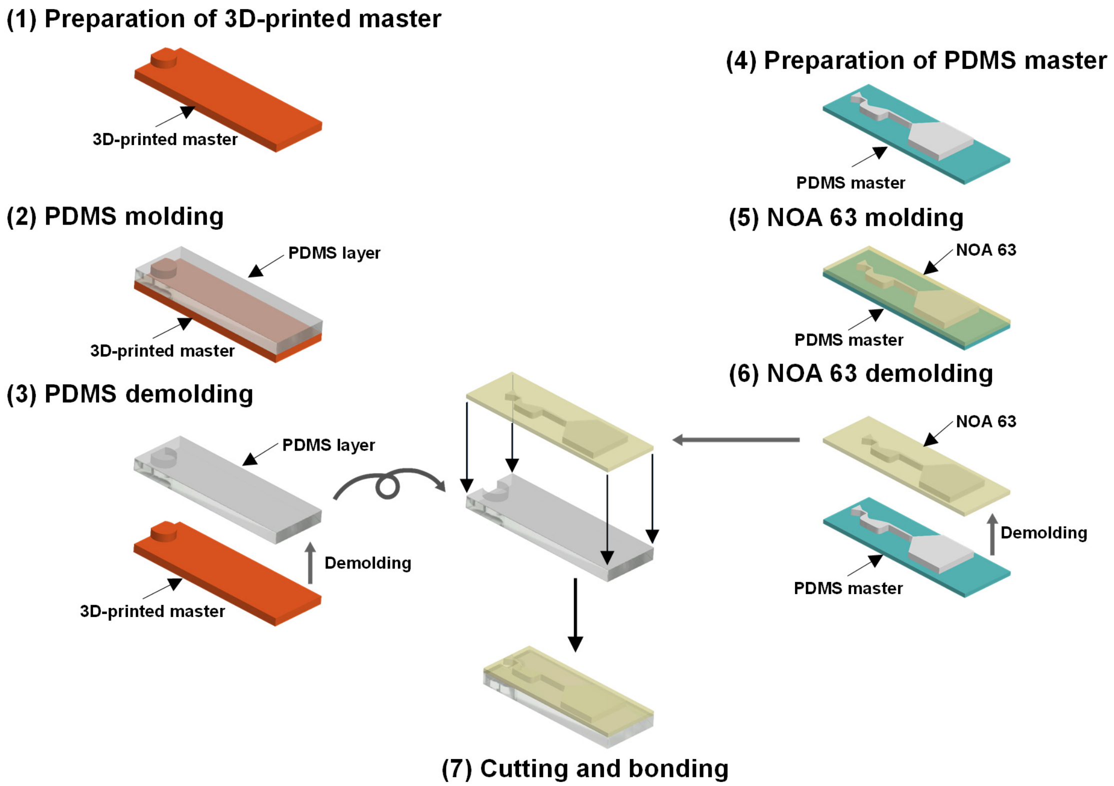

Fig. S4. The fabrication process of the microfluidic chips. (**1**) Preparation of the 3D-printed master. (**2**) PDMS layer prepared by casting on the 3D-printed master (**3**) PDMS layer curing and demolding. (**4**) PDMS master prepared by casting. (**5**) NOA 63 molding. (**6**) UV curing and demolding of NOA 63. (**7**) Bonding between NOA 63 and PDMS layer after plasma treatment.

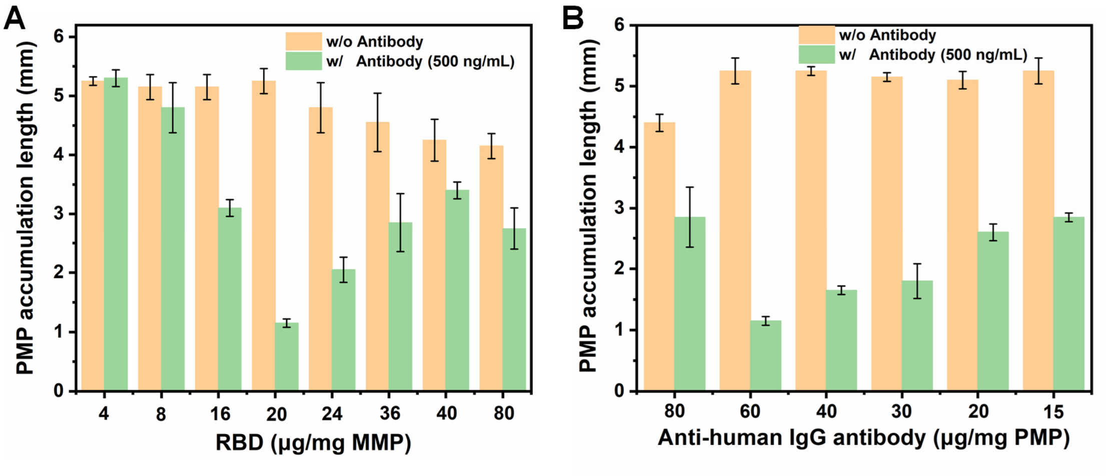

Fig. S5. Optimization of protein immobilization of microparticles for detection of anti-spike RBD IgG in undiluted serum/plasma using microfluidic chips. Optimization of (**A**) RBD on MMPs (20 mg/ml of PMPs, 5 mg/ml of MMPs, 40 μg of anti-human IgG anibody per milligram of PMPs), (**B**) anti-human IgG antibody on PMPs (20 mg/ml of PMPs, 5 mg/ml of MMPs, and 20 μg of RBD per milligram of MMPs) for detection on the microfluidic chip (mean ± SD, n = 3).

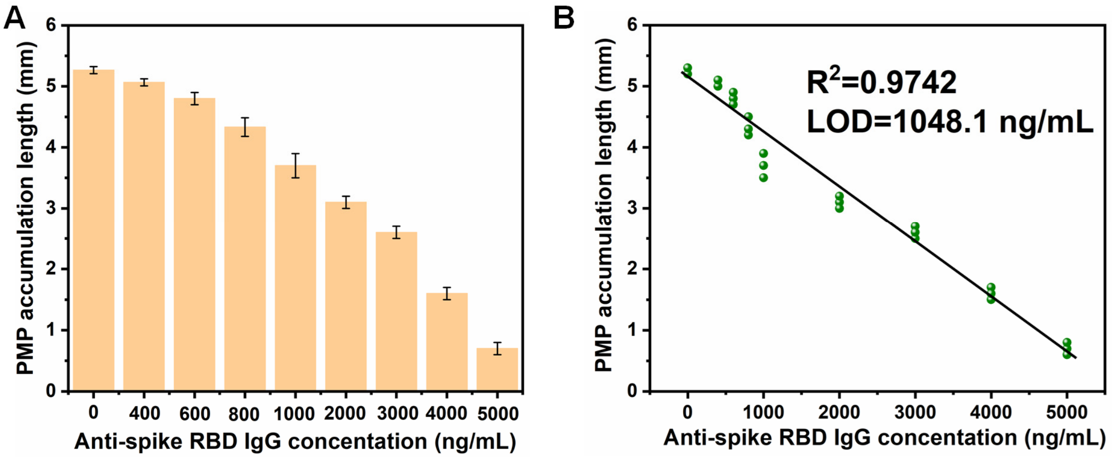

Fig. S6. Detection of anti-spike RBD IgG in undiluted whole blood. (**A**) The measured PMPs accumulation length with respect to varied concentrations of anti-spike RBD IgG in whole human blood using rapid mode, (mean ± SD, n = 3). (**B**) The linear regression of (A) from 0 to 5,000 ng/mL showing a linear equation as $y_{(0-5000\ \mathrm{ng/ml})} = 5.1343 - 0.0009x \pm 0.414\left(\frac{10}{27} + \frac{(x-186.67)^2}{20862292}\right)^{\frac{1}{2}}$.

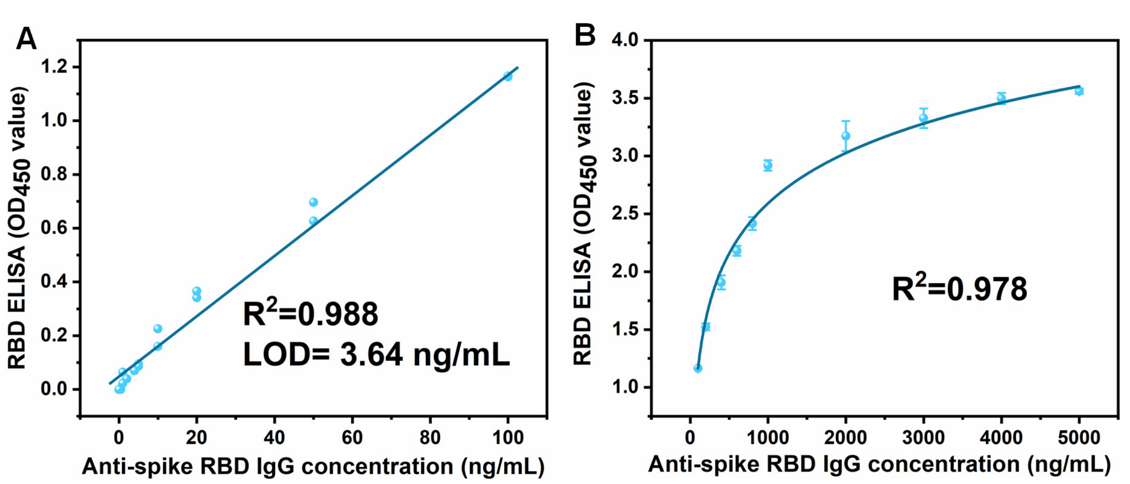

Fig. S7. Standard curve of ELISA based on anti-spike RBD IgG spiked in human serum. (**A**) For concentration 0 to 100 ng/ml, the linear regression equation: $y_{(0-100\ \mathrm{ng/ml})} = 0.0368 + 0.0117x \pm 0.0146\left(\frac{23}{60} + \frac{(x-19.35)^2}{385}\right)^{1/2}$. (**B**) For concentration 100 to 5000 ng/mL, the non-linear regression equation: $y_{(100-5000\ \mathrm{ng/ml})} = 0.6509\ln x - 1.8791 \pm 0.64\left(\frac{23}{60} + \frac{(\ln x-6.83)^2}{136.6}\right)^{1/2}$.

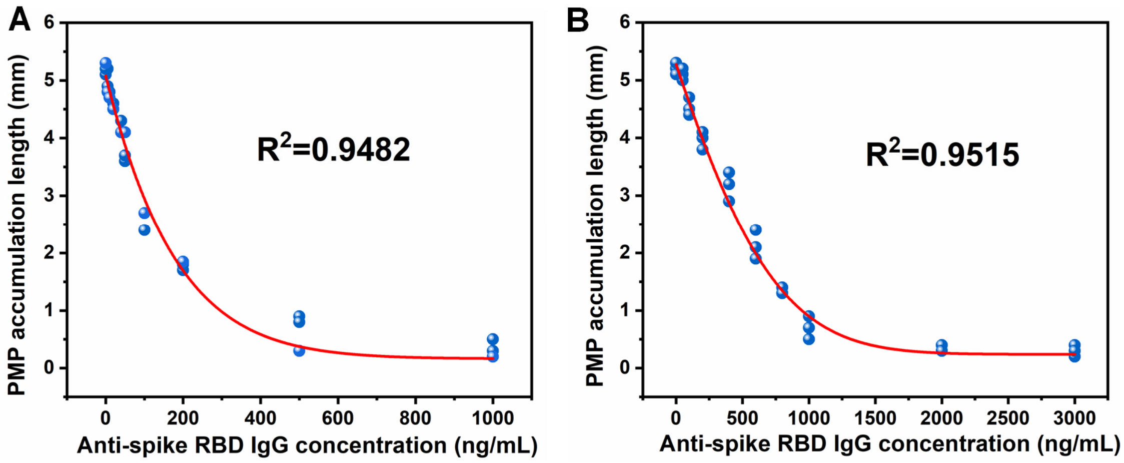

Fig. S8. Standard curve of microfluidic chips based on anti-spike RBD IgG spiked in human serum. (**A**) Sensitive mode, the non-linear regression equation: $y_{(5-1000\ \mathrm{ng/mL})} = -0.984\ln x + 7.1683 \pm 0.733\left(\frac{10}{27} + \frac{(\ln x - 4.17)^2}{26.37}\right)^{1/2}$. (**B**) Rapid mode, the non-linear regression equation: $y_{(50-3000\ \mathrm{ng/mL})} = -1.342\ln x + 10.657 \pm 1.186\left(\frac{10}{27} + \frac{(\ln x - 6.16)^2}{39.614}\right)^{1/2}$.

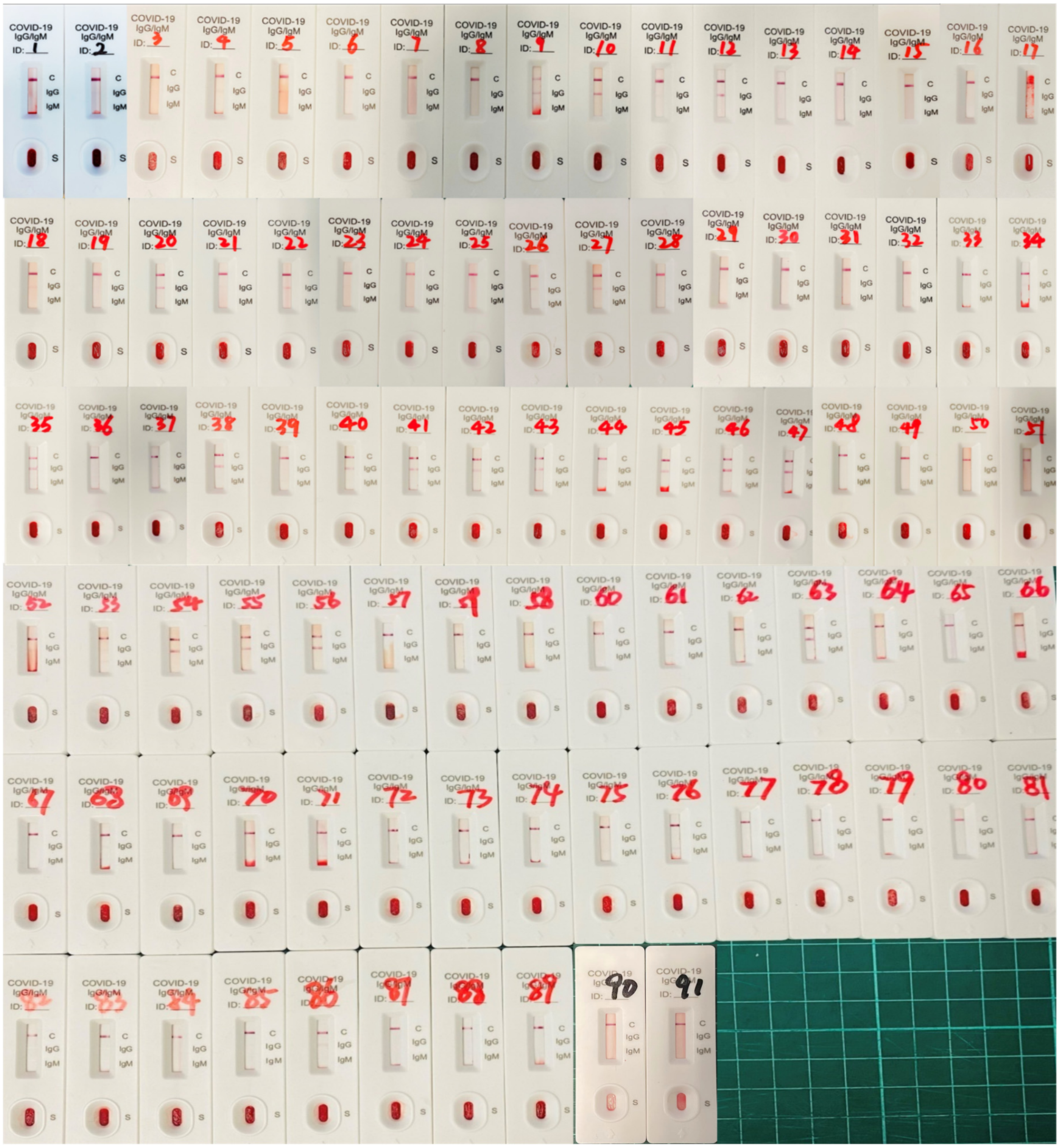

Fig. S9. Photos of LFIA test results. Results from fingerprick whole blood samples of the 91 vaccinees using COVID-19 Rapid Test Kit IgG+IgM (Wuhan UNscience Biotechnology Co., Ltd).

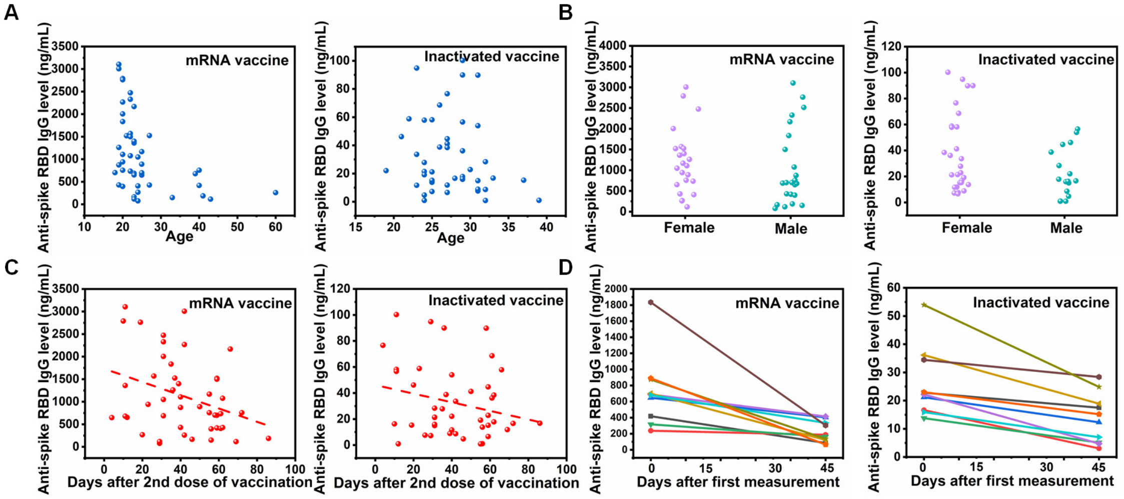

Fig. S10. Level of anti-spike RBD IgG based on age, gender, and time by ELISA. (**A-C**) Level of plasma anti-spike RBD IgG based on age (A), gender (B), and days from the second dose of vaccine (C). The dashed line in (C) is the linear regression. (**D**) Anti-spike RBD IgG level of 20 vaccinees of the first measurement and on the 45th days after the first measurement.

Table S1 The sensitivity of LFIA.

Vaccine type	No. Volunteers (n)		Positive	Equivocal	Negative	Sensitivity (equivocal excluded)
**mRNA vaccine**	46		1	2	43	6.52%
**Inactivated vaccine**	45		15	6	24	46.67%

Table S2. The acronyms and the corresponding full names.

Acronyms	Full names
MMPs	Magnetic Microparticles
PMPs	Polystyrene Microparticles
LOD	Limit of Detection
COVID-19	Coronavirus Disease 2019
WHO	World Health Organization
IgG	Immunoglobulin G
IgM	Immunoglobulin M
SARS-CoV-2	Severe Acute Respiratory Syndrome Coronavirus 2
ELISA	Enzyme-Linked Immunosorbent Assay
LFIA	Lateral Flow Immunoassay
RBD	Receptor Binding Domain
anti-spike RBD IgG	IgG Antibody against SARS-CoV-2 Spike Protein Receptor-Binding Domain
anti-human IgG	Secondary Antibody against Human IgG
anti-spike IgG	IgG Antibody against SARS-CoV-2 Spike Protein
ACE2	Angiotensin-converting Enzyme 2
UV-Vis	Ultraviolet-visible
NOA 63	Norland Optical Adhesive 63
MERS-CoV	Middle East Respiratory Syndrome Coronavirus
HCoV-HKU1	Human Coronavirus HKU1
PDMS	Polydimethylsiloxane
SLIP-LAB	Slippery Liquid-Infused Porous Surface Laboratory
OD450	Optical Density at the Wavelength of 450 nm
MES	2-(N-Morpholino)ethanesulfonic Acid
EDC	1-ethyl-3-(3-dimethylaminopropyl)carbodiimide Hydrochloride
NHS	N-hydroxysuccinimide
PBS	Phosphate Buffered Saline
BSA	Bovine Serum Albumin
APC	Allophycocyanin
TNT	Tris (Tris(hydroxymethyl)aminomethane), NaCl (sodium chloride), and Tween 20

Movie S1. Demonstration video of the operation procedure using the rapid mode. MMPs coated with RBD are first mixed with undiluted serum/plasma for 5 min with handshaking to specifically capture the anti-spike RBD IgG. After rinsing using a magnetic rack, the interfering materials such as other human antibodies can be removed before incubating with PMPs coated with anti-human IgG antibody for another for 5 min with handshaking. Next, the solution is dispensed into the loading chamber, and the device is placed vertically. Subsequently, the solution enters the microchannels in the NOA layer and forms PMP accumulation in the trapping channel visible to the naked eye within 10 min.

Movie S2. Magnetic separation of MMPs and PMPs with the presence of anti-spike IgG. As PMPs-antibodies-MMPs are formed, less free PMPs can flow and be trapped at a particle dam. Thus, the presence of anti-spike IgG shortens the PMP accumulation length.

Movie S3. Magnetic separation of MMPs and PMPs without the presence of anti-spike IgG. As MMPs and PMPs are not connected, almost all PMPs are allowed to flow and trapped at a particle dam, causing a longer PMP accumulation length.

Movie S4. PMP accumulation as a monolayer in the trapping channel. The PMPs accumulate into a monolayer in the channel with height of 25 ± 0.3 μm, which maximizes the visibility of PMP accumulation length.

Data processing and analysis

- **Linear regression**

A linear model, $y = b_0 + b_1 x$, is chosen to perform least-squares regression, where y represents the result of assays, i.e., optical absorbance, trapping length of the PMP accumulation, or OD_{450} value, and x represents the concentration of SARS-CoV-2 antibody. In the linear regression, the estimates of the intercept b_0, the slope b_1, their variances $s_{b_0}^2$, $s_{b_1}^2$ and the residual variance of the regression $s_{\frac{y}{x}}^2$ is determined by:

$$b_0 = \bar{y} - b_1 \bar{x} \quad (1)$$

$$b_1 = \frac{\sum_{i=1}^{n}(x_i - \bar{x}) y_i}{\sum_{i=1}^{n}(x_i - \bar{x})^2} \quad (2)$$

$$s_{b_0}^2 = s_{\frac{y}{x}}^2 \left(\frac{1}{n} + \frac{\bar{x}^2}{\sum_{i=1}^{n}(x_i - \bar{x})^2}\right) \quad (3)$$

$$s_{b_1}^2 = \frac{s_{\frac{y}{x}}^2}{\sum_{i=1}^{n}(x_i - \bar{x})^2} \quad (4)$$

$$s_{\frac{y}{x}}^2 = \frac{\sum_{i=1}^{n}(y_i - \hat{y}_i)^2}{n-2} \quad (5)$$

where n is the total number of data point calculated by $n = \sum_{j=1}^{k} m_j$, k is the number of concentration levels, m_j is the times of repetition at each concentration level, $\bar{x}$ and $\bar{y}$, is the mean value of x and y, as shown below:

$$\bar{x} = \sum_{i=1}^{n} \frac{x_i}{n} \quad (6)$$

$$\bar{y} = \sum_{i=1}^{n} \frac{y_i}{n} \quad (7)$$

and $\hat{y}_i$ is the predicted value of y for a particular concentration x_i, as shown in equation:

$$\hat{y}_i = b_0 + b_1 x_i \quad (8)$$

Hence, the calibration curve is expressed as:

$$y = b_0 + b_1 x \pm t_{(\alpha, n-2)} s_{\frac{y}{x}} \left(\frac{1}{m} + \frac{1}{n} + \frac{(x - \bar{x})^2}{\sum_{i=1}^{n}(x_i - \bar{x})^2}\right)^{\frac{1}{2}} \quad (9)$$

where $t_{(\alpha, n-2)}$ is the critical value of student t distribution, which is selected as 1.645 for the 90% confidence interval of two-tailed hypothesis ($\alpha = 0.05$), and *1/m* is the contributes of uncertainty from the average of *m* replicates in future observation (*46, 48*).

- **Limit of detection**

For the estimation of the limits of detection x_D, a non-central t-distribution model is selected with the equation:

$$x_D = \delta_{(\alpha, \beta, n-2)} \frac{s_{\frac{y}{x}}}{b_1} \left(1 + \frac{1}{n} + \frac{\bar{x}^2}{\sum_{i=1}^{n}(x_i - \bar{x})^2}\right)^{\frac{1}{2}} \quad (10)$$

where $\delta_{(\alpha,\beta,n-2)}$ is the non-centrality value of the non-central t-distribution taking protection against both type I error rate (α, false-positive) and type II error rate (β, false-negative) (*46, 48*). All LOD was determined based on an appropriate linear range evaluated by the R^2.

- **Accuracy**

The measured antibody level by microfluidic chip (*Y*) is compared with that by the gold standard method ELISA (*X*) to study the accuracy. The agreement between two sets of measured antibody levels of the 91 volunteers' plasma sample is quantified using Lin's concordance correlation coefficient ($\hat{\rho}_C$), as shown below (*55*):

$$\hat{\rho}_C = \frac{2\rho\sigma_Y\sigma_X}{(\mu_Y-\mu_x)^2+\sigma_Y^2+\sigma_X^2} \quad (11)$$

where μ_Y and μ_x are the means, σ_Y^2 and σ_X^2 are the variances, and ρ is Pearson's correlation coefficient.
